# Factors influencing sexual and reproductive health of Muslim women: a systematic review

**DOI:** 10.1186/s12978-020-0888-1

**Published:** 2020-03-05

**Authors:** Noura Alomair, Samah Alageel, Nathan Davies, Julia V. Bailey

**Affiliations:** 1 Research Department of Primary Care and Population Health, Institute of Epidemiology and Health Care, University College London, Upper 3rd Floor, Royal Free Campus, Rowland Hill Street, London, NW3 2PF UK; 2 Community Health Sciences Department, College of Applied Medical Sciences, King Saud University, Riyadh, Kingdom of Saudi Arabia

**Keywords:** Reproductive health, Sexual health, Sex education, Contraception, Family planning, Islam, Culture, Religion, Women health, Systematic review

## Abstract

**Background:**

In Islamic societies, issues related to sexual and reproductive health (SRH) are rarely discussed and considered sensitive subjects. This review aimed to identify any personal, religious, cultural, or structural barriers to SRH service and education among Muslim women worldwide.

**Methods:**

A search for qualitative and quantitative studies was conducted on seven electronic databases. A narrative synthesis using thematic analysis was conducted.

**Results:**

Fifty-nine studies were included from 22 countries: 19 qualitative, 38 quantitative and two mixed methods. Many Muslim women have poor SRH knowledge, and negative attitudes which influence their access to, and use of SRH services. Barriers to contraception use among Muslim women included a lack of basic reproductive knowledge, insufficient knowledge about contraception, misconceptions, and negative attitudes. Women had negative attitudes towards family planning for limiting the number of children but not for child spacing, which reflected religious views towards family planning. Religious and cultural beliefs were barriers to contraception use and access to SRH services and information. Family and the community have a significant impact on women’s contraceptive use and access to SRH services. Husband and family opposition played a significant role in contraception access and use. Fear of stigmatization and being labelled as having pre-marital sexual relations among unmarried women acted as the main barrier to accessing contraception and seeking SRH information and services.

**Conclusion:**

The findings reveal that there are multiple levels of factors that influence Muslim women’s SRH. Poor SRH knowledge and practices among Muslim women is complex matter that is affected by personal, community, cultural, religious factors and existing policies and regulations. All these factors overlap and are affected by each other. There is an urgent need for interventions addressing modifiable barriers to SRH education and services to improve knowledge, informed choice and access to services to facilitate better sexual and reproductive wellbeing for Muslim women. It is important to note that while this review aimed to report findings on Muslim women, we acknowledge that significant variations exist within every culture and religion.

## Plain English summary

Issues related to sexual and reproductive health (SRH) are rarely discussed in Muslim societies. This could result in poor SRH knowledge and practices among Muslim women. The study aimed to identify factors influencing Muslim women access and use of SRH education and services worldwide through systematic review of published scientific articles.

We identified 59 studies from 22 countries to be included in this review. The study identified personal, family and community, cultural and religious and health policy and health services factors to influence Muslim women’s SRH. Lack of knowledge about family planning methods and available SRH services was reported as a key barrier. Women’s attitude towards family planning was influenced by religious views towards contraception. Many Muslim women accepted the use of family planning for child spacing, but believed it was unacceptable to limit the number of children. Women’s control over SRH choices was restricted by oppositions of family members, especially the husband and mother in-law. Cultural unacceptability of family planning influenced women’s ability to access SRH education and services. Unmarried women face greater difficulties accessing SRH services due to the cultural belief that unmarried women should not be sexually active and should not be provided with SRH information and services. The attitudes and skills of healthcare providers sometimes affected Muslim women’s access and use of SRH education and services. Barriers included healthcare provider’s misinformation, lack of skills, judgemental and disrespectful treatment.

There is a need to develop strategies to facilitate Muslim women’s access to SRH services. The provision of culturally appropriate SRH education could have a positive impact on women’s knowledge and access to SRH services, consequently leading to overall improvements in women’s SRH.

## Introduction

Religion and culture can play an important role in the education and lifestyle of its followers, and this is clearly observed in Islamic societies where certain social behaviours are prohibited or considered to be unacceptable, such as extra-marital sexual relations. Issues related to sexual and reproductive health (SRH) are rarely discussed in Muslim societies and are considered sensitive subjects [1, 2]. Failure to provide SRH education may result in serious health threats including unwanted pregnancy, unsafe abortion and sexually transmitted infections (STIs) [3]. Teenage pregnancies and unsafe abortions all contribute to morbidity and mortality, with girls aged 15–19 years twice as likely to die from childbirth as women in their twenties worldwide [4].

In Islamic cultures, there is a widespread assumption that single women do not need to be knowledgeable about their own SRH [1, 2, 5, 6]. This assumption stems partly from the high value placed by society on women’s virginity before marriage, and the belief that talking about SRH might encourage pre-marital sexual relations. Yet a number of reviews of sex education programmes worldwide suggest that such programmes could lead to delayed first intercourse, contraceptive use and safer sexual practices [7, 8]. Literature also suggests that in some Islamic societies, unmarried women are less likely to be referred for reproductive health services [9] and are also less likely to seek reproductive healthcare than married women [10]. Lack of SRH services makes Muslim women, both married and single, a vulnerable group unable to make or act on informed decisions about their own reproductive health.

A number of systematic reviews have covered many aspects of women’s SRH; however, none have focused exclusively on Muslim women, and the factors affecting their SRH. In the United Kingdom (UK) and United States, ethnic variations in SRH service utilization and outcomes have been documented and are considered to be a major public health concern [11–13]. For example, compared to White women, women of Asian origins are less likely to attend sexual health clinics and less likely to use emergency contraception [11] and are more likely to delay antenatal care [13]. Ethnic differences in SRH existed even after adjusting for possible explanatory factors, such as socioeconomic status and sexual behaviour [11]. However, these studies often fail to take into account the wider cultural and religious determinants of SRH. It is essential to understand the factors that influence Muslim women’s access and use of SRH services and education. Therefore, this systematic review aimed to identify any personal, religious, cultural, or structural barriers to SRH service and education among Muslim women. In particular, the review focused on knowledge, attitudes, experiences, and healthcare-seeking behaviours in issues related to SRH.

## Methods

### Design

A systematic literature search for qualitative and quantitative studies was conducted following the Centre for Reviews and Dissemination guidelines [14]. A narrative synthesis approach with thematic analysis methods was used in this review. The protocol for this systematic review was registered with PROSPERO (registration number: CRD42017081999).

### Search strategy

Seven electronic databases were searched with a tailored search strategy for each database, searching from 2007 to January 2017, updated in February 2018. These were; MEDLINE, EMBASE, WEB OF SCIENCE, PsycINFO, Maternity & infant care, CINAHL, and POPLINE. A further screening was done on reference lists of all included papers for additional studies. The search strategy combined terms for sexual health OR reproductive health AND Muslim women. The full search strategy of MEDLINE database can be found in Additional file 1.

### Inclusion criteria

Included were studies published between 2007 and 2018, published in English or Arabic. The search focused on studies of Muslim women of reproductive age (or studies that presented Muslim females’ results separately from male/non-Muslim results) and used qualitative or quantitative study designs.

Muslim women were defined as women who identified themselves as Muslims or who live in countries where the dominant religion is Islam. Muslim countries were defined as countries where over 90% or more of the population are Muslims. Sexual and reproductive health was defined in this review as SRH information, education and services, including contraceptive knowledge, attitude and use.

### Exclusion criteria

Systematic reviews, intervention studies, studies in the form of editorials, conference abstracts, and policy documents were excluded.

### Selection procedure

Two reviewers (NA & SA) independently reviewed all citations. After the title screening stage was complete, the two datasets produced by each reviewer were combined. The same steps were taken for abstract/full-text screening results. All disagreements were resolved by consensus and disagreements were discussed to determine if the citation included the information needed.

### Data extraction

A standardised data extraction form was developed to identify participant characteristics, study design, aims, data collection methods, data analysis method and main findings for all included studies. One reviewer (NA) extracted all data from all included full texts. Second reviewer (SA) reviewed a random selection of 50% of the extracted data. All disagreements were resolved by consensus. Third and fourth reviewers (JB & ND) intended to resolve any remaining disagreement. If reviewers required more information, authors were contacted.

### Quality assessment

For this review, the quality of studies with qualitative design was assessed using the Critical Appraisal skills programme [15]. For appraising the quality of quantitative studies, we used the quality assessment tool developed by the Centre for Evidence Based Management [16]. Both tools have been widely used to assess the quality of qualitative and quantitative evidence. One reviewer (NA) assessed the quality of all included studies and a second reviewer (SA) assessed 50% of the studies. Third and fourth reviewers (JB & ND) intended to resolve any remaining disagreement.

### Data analysis

The data in this review were synthesised using Popay et al. (2006) narrative synthesis approach [17]. It allows us to combine the results of qualitative and quantitative studies and examine relationships between them. Since both qualitative and quantitative studies were included in this review, the data were translated into common categories/headings to allow for useful comparisons of the results.

**Fig. 1:**
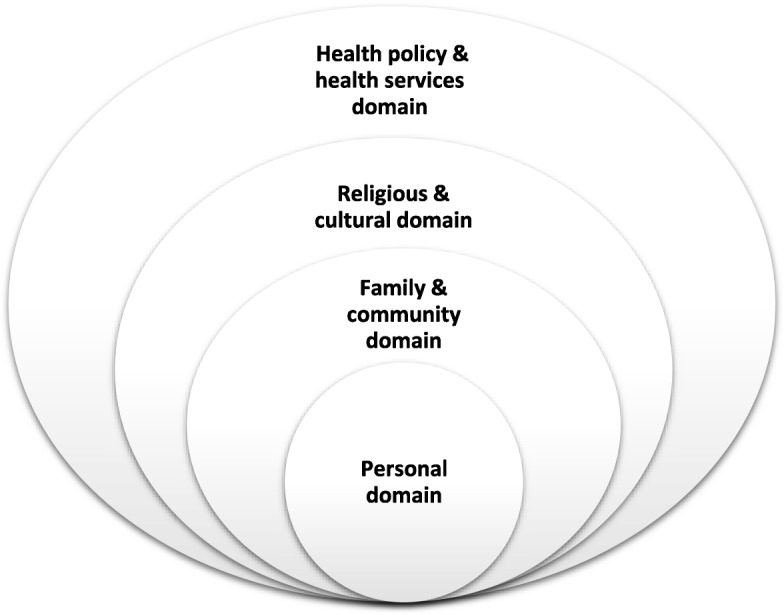
Conceptual framework guiding the analysis of the barriers and facilitators to reproductive health of Muslim women

After completing the thematic analysis, the themes of both qualitative and quantitative studies were merged and tabulated, and the first draft of the conceptual framework (Fig. 1) was developed and discussed by the review team. The conceptual framework used for the review was based on a modified version of the ecological model of health behaviours [18]. Discussions resulted in the rearrangement of themes, producing the final conceptual framework. The framework recognises that multiple levels of factors influence health behaviours. These were divided into: Personal domain (knowledge, attitudes, skills), Family and Community domain (friends, family, social networks), Religious and Cultural domain (religious beliefs, and sociocultural norms), and Health Policy and Health Services domain (national, state, local laws and regulations).

## Results

### Study characteristics

**Fig. 2:**
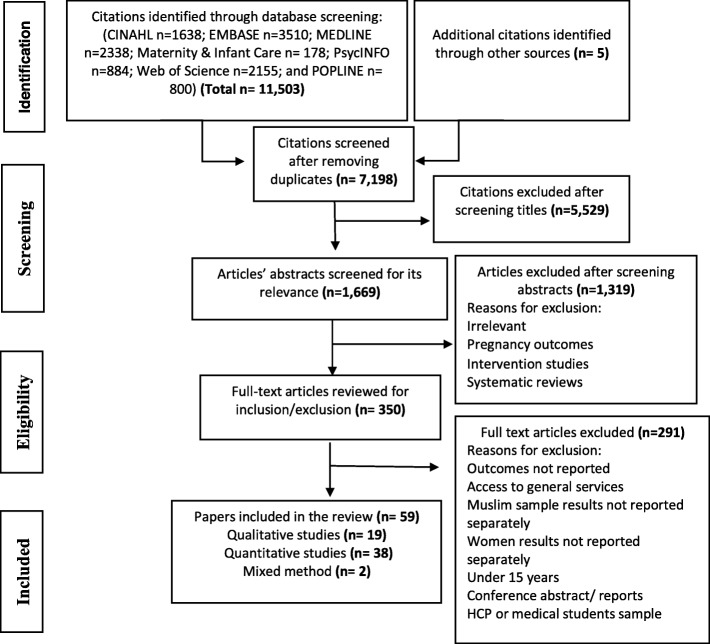
PRISMA flow diagram outlining the systematic review process

The search identified 11,503 articles, and five additional records from article reference lists. All duplicates were removed, leaving 7198 articles for title screening. After title screening, 1669 abstracts were screened to identify potentially relevant studies. Finally, 59 studies met the inclusion criteria and were included in the review. Figure 2 shows the Preferred Reporting Items for Systematic Reviews and Meta-Analyses (PRISMA) flow diagram outlining the systematic review process.

### Study design and sample characteristics

The results of 59 published papers were synthesised. Thirty-eight studies were cross-sectional surveys [19–71], nineteen were qualitative studies [72–90], and two employed mixed methods design [91, 92]. Table 1 shows a list of the included studies, giving details of settings, instruments and outcomes.

**Table 1:** Summary of study characteristics

Characteristic	Number of studies *N* = 59
Study design	
Quantitative	38
Qualitative	19
Mixed method	2
Country	
Turkey	12
Iran	10
Saudi Arabia	6
Egypt	5
Jordan	5
Afghanistan	3
India	3
Other	15
Data collection/instrument	
Self-administered questionnaire	7
Interview questionnaire	27
Semi-structured/ in-depth interviews	10
Interview questionnaire +semi-structured interviews	1
Focus groups	5
Interviews + focus groups	4
Ethnography + interviews	1
Not reported	4
Outcomes reported	
Contraception knowledge, attitudes, access and use	48
Sexual and reproductive health education and services knowledge, attitudes, access and use	11

The majority of studies were conducted in Iran and Turkey, which are the fastest growing medical research output in the Middle East [93]. The majority of studies (*n* = 48) focused on factors related to contraception knowledge and use. Of the 48 studies focusing on contraception, almost all studies (*n* = 46), exclusively included married women in their sample, with some specifying ‘currently married’ rather than ‘ever married’ in the eligibility criteria. Some authors have justified that it is socially unacceptable to question unmarried Muslim women about contraception, as the general assumption is that they are not sexually active [36, 50, 52, 90]. The majority of women in the included studies were unemployed ‘housewives’ [19–21, 23, 25–27, 29, 35, 37–39, 46, 47, 49–51, 91], and a significant proportion were illiterate or had education no more than secondary school education [19–21, 23–29, 34–38, 40–43, 45–47, 49, 50, 91], with one study from Afghanistan reporting illiteracy levels as high as 92% [40]. Interview questionnaire was the most commonly used data collection method, which is suitable for participants with low literacy.

### Methodological quality

Overall, studies were of good to moderate quality. Thirty-five studies were judged to be of good quality [20–22, 26, 27, 30, 31, 34, 37, 38, 40, 42, 44, 49, 50, 52, 72–78, 81–92]. These studies had a clearly focused research aim and the research methodology was judged to be appropriate. Twenty two studies were of moderate quality [19, 23–25, 28, 29, 32, 33, 35, 36, 39, 41, 43, 45–48, 51, 53, 79, 80]. Two studies only were of poor quality [68, 70]. Sixteen studies included failed to clearly describe how participants were selected [19, 24, 28, 29, 33–35, 48, 51, 52, 56, 57, 59, 61, 70], and twenty-two studies used sampling strategies that had the potential to introduce selection bias [19, 22, 23, 25, 29, 32, 34, 37, 39, 43, 45–48, 50–52, 55, 59, 62, 63, 67, 71, 91]. Only 26 studies appeared to have a sample representative of the target population [20, 26, 27, 30–34, 36, 38, 40–42, 44, 46, 49, 53, 54, 58, 60, 64–69, 91]. Only 16 studies reported on response rate [19, 20, 23, 26, 27, 38, 44, 49, 50, 53, 58, 62–66], and one study had a low response rate of 50% [63]. Some qualitative studies provided a combination of healthcare professionals’/policy makers’/ religious leaders’/ husbands ‘views in addition to women’s views; and in certain instances, it was difficult to distinguish the views of women from other participants in the sample.

The findings of this review are presented in themes that fall under one or more of the conceptual framework domains (Fig. 1). Where available, women’s marital status and age are presented along with their quotes.

#### Theme 1: Insufficient knowledge and misconceptions about contraception and reproductive health services

From the personal domain, knowledge about family planning methods and available health services was discussed as a factor influencing women's SRH [19–21, 25, 26, 28, 30, 32, 33, 35, 39, 43–45, 47–53, 68, 70, 77, 79, 80, 83, 86, 87, 89, 90]. Insufficient knowledge about contraception and lack of basic reproductive knowledge was reported as one of the main barriers to contraception use among Muslim women [20, 26, 29, 31, 39, 41, 46, 83, 90, 91]. Some women are unaware of services available for them [69, 74, 75, 78, 87], particularly unmarried women [74, 75, 78].

*“Giving information is more important than the services, as many do not know what services are provided in healthcare centres and whether the services can be used for singles or not. I, too, do not know where I can go. I just know that there are gynaecologists, and the health centre is only for married women.”* **Unmarried woman, age 29 – Iran** [74]

Misconceptions about the side-effects, modes of action, or effectiveness of different contraception methods also contributed to poor uptake and use among women [19, 21, 24, 26, 29, 31, 33, 35, 38, 39, 41, 47, 50–52, 68, 80, 81, 86, 89–91]. Reported misconceptions included the belief that contraception could lead to infertility, difficulty and pain during intercourse, cancer, early menopause, hair growth, mental health problems, doubts about methods reliability with some women believing that the risk of pregnancy is high with the oral contraceptive pill and others believe that intrauterine devices can travel within the body [21, 35, 47, 48, 51, 52, 80, 81, 86, 89, 90].

*“I heard that a coil was attached to the baby, it [IUD] was found somewhere on the baby. I was scared, I had an ultrasound. They said that it [my IUD] was in place. Still, I was scared, so I wanted it to be taken out.”* **Married woman – Turkey** [80]

#### Theme 2: Sources of information on contraception

Main sources of information included family, neighbours, friends, media, healthcare professionals, books and television [32, 33, 49]. Qualitative evidence suggests that information on family planning was predominantly obtained from family, neighbours and friends [80–83, 86, 89, 90].

*“When women gather, they talk about these things [family planning] so I know about those things from neighbours and family … my sister told me about condoms and we started to buy condoms afterwards.”* **Married woman – Jordan** [90]

Only in two studies conducted in Australia and the UK, women mentioned education in school as a source of family planning information. However, in the UK study, women felt that amount of information received from schools was inadequate [76, 87].

#### Theme 3: Barriers to sexual and reproductive health education and information needs

Barriers to SRH education were influenced by multiple domains of the conceptual framework. Personal attitudes were affected by religious beliefs, in addition to family and community factors. Some women exhibited negative attitudes towards SRH education and felt that they do not need to be educated about SRH matters as their religious practices provided them with enough protection against STIs [72, 75, 76].

*“We don’t go through the process of multiple partners. That is a religious distinction. So far now, grace of God. It keeps us safe from many sicknesses. The rest, Allah’s wisdom is used. At least we have kept ourselves safe from sexually transmitted disease.”* **Woman, marital status unknown – Canada** [72].

Others thought of SRH education as encouraging early sexual relations among unmarried youth [70, 76, 78]. In some instances, it was thought to be against religious beliefs to teach SRH topics to unmarried youth [76, 78]. Yet, many women believed it is essential to learn about SRH, with some emphasising the preference of being taught in a structured manner and delivered by trained professionals. It was also highlighted by many women that the content of SRH education needs to be tailored to fit Muslim women’s needs [69, 73, 75, 76, 78, 79].

*“There needs to be more support for Muslim women who are not too sure about sex and Islam. It can be hard to know exactly what is right and wrong sometimes, like where the line is. Also, I don’t know too much about sexual health, so I think it would be good to learn this with other Muslim women.”* **Unmarried woman, age 25 – Australia** [76].

One study interviewed religious leaders and asked for their views on providing SRH education for unmarried youth. Most of them had supporting views on the importance of SRH education in Islam and considered that it was not against Islamic beliefs. They did however emphasise the importance of having an education within the boundaries of Islamic teachings and providing culturally and religiously sensitive content [78].

*“People who think that sex education isn’t permitted in religion are completely wrong, but such knowledge should be taught in a way that informs adolescents about sexuality in a modest and moral manner.”* **Religious leader, age 48 – Iran** [78].

Findings suggested that mothers’ attitudes towards SRH education affected young girls’ access to information and education [77–79]. Many mothers wanted their daughters to learn but felt that they had inadequate information to give them, and others were embarrassed discussing such matters with their daughters [70, 78].

*“Our information about adolescents’ sexuality is low; it’s better the necessary information is taught to them [our daughters] in school.”* **Married woman, age 46 – Iran** [78]*.*

#### Theme 4: Socio-demographic factors influencing contraceptive use

Several personal factors were associated with contraceptive uptake and use. Age, education, employment, and number of children were all found to play a significant role in predicting contraception use among women [21, 23–27, 29, 33–38, 40–44, 91]. An increase in women’s age was associated with increased contraceptive use [21, 25, 27, 29, 34, 38, 40, 42]. Women in their late 20s and 30s were more likely to use contraception than younger age groups (15–25 years). Parity was significantly associated with women’s contraceptive use [21, 24, 33, 36, 37, 40–44], with contraceptive use increasing with a rising number of children [24, 33, 36, 37, 40–43]. One study conducted in Thailand showed opposing findings, with smaller numbers of children associated with more contraceptive use. The authors concluded this was the result of women in their sample having low income and wanting to restrict the number of children in order to maintain a good quality of life [44]. The educational level of both women and their husbands significantly influenced their use of contraception. Women’s and husbands’ education was positively associated with use [21, 23, 24, 27, 29, 33, 34, 36, 37, 40–43]. With regards to employment, working women use contraception more frequently than non-working women [21, 23, 27].

#### Theme 5: Attitudes towards family planning

The majority of women had positive attitudes towards family planning for child spacing [19, 21, 26, 28, 30, 32, 33, 36, 39, 76, 79–81, 86, 90]. Several benefits of family planning for child spacing were mentioned including economic benefits, better health outcomes for the mother and child, leading to overall improvements on the quality of life [19, 81, 84, 86, 89, 90].

*“I think it would be better if I had planned births actually, because I want to have better health for myself and to take good care of my children.”* **Married woman – Jordan** [90].

On the other hand, many women expressed negative attitudes towards family planning for limiting the number of children [26, 32, 39, 86, 88, 89]. Some women believed that it would be easier to raise and care for their children if they were closely spaced.

*“If I give more space, I face a lot of difficulties; short space is good. I can feed them in one time, they can play together, and they can go to school together. Long space creates more problems. This is my experience.”* **Married woman – Afghanistan** [81].

There were context-specific reasons for wanting to have many children in countries like Afghanistan, where child mortality is high, or in countries affected by war and conflict like Syria and Palestine [81, 88, 90].

*“For me and women in Palestine, we like to have more children because many of the kids get arrested or shot by the Israelis, so we like to have more children – we don’t want to lose all of our children. But at the same time it’s really hard for us because of the economic situation and poverty”* **Married woman, age 32 – Palestine** [88]*.*

Different interpretations of the economic impact of child spacing was found in one study [81], where women felt that having many children was an economic advantage.

*“[Many women are] interested in a lot of children because when they grow up, they will earn money for them.”* **Married woman – Afghanistan** [81].

It was observed that some women had negative attitudes towards condoms use, based on their perception that they are only used to protect against STIs in case of extra-marital sexual relations [80].

*“God forbid, the men who go out [who sleep with other women] use it [condoms]. I did not hear it from the doctor; I had heard it from the television. The risk of disease decreases, that is what they had said.”* **Married woman – Turkey** [80].

#### Theme 6: Women’s lack of control over reproductive choices

According to Muslim women, family and community factors had a crucial impact on their decision to use family planning methods. Husbands’ opposition was cited as one of the main barriers to contraception, and it was observed that the husband is the key decision maker about family planning [19, 24, 26, 31, 33, 35, 38, 39, 41, 52, 68, 81, 83–92]. Decision-making is not usually shared, and, in some cases, women have no control over their own fertility. The main reason for husbands’ refusal was their desire to have more children, in some cases, specifically sons [84, 86, 88, 90, 92].

*“I may get pregnant, but I’m consoling myself. I breastfeed day and night. All people get sleep at night except me still feeding my child. I get tired, but what to do? My husband is not allowing me to use any modern method”* **Married woman, age 33 – Jordan** [86].

Men are generally perceived to be indifferent or unaware of their wives’ reproductive health needs; and women are traditionally expected to be shouldering contraceptive responsibility alone. Although husbands were often the primary decision makers for contraception use, they rarely wanted to share accountability for preventing pregnancies [81, 83, 86, 87, 91].

*“When one participant handed her husband condoms saying, “I don’t want to bear a child. You also don’t want. Our children are still small. You do nothing. Use these at least,” he said, “Are you joking with me?! You do it!”* **Married woman, age 31 – Turkey** [83].

Since polygamy is permissible in Islam, some women considered having many children to be key in keeping the stability of their marriage and sustaining the husband’s attention and financial support [85, 88, 92]. Some husbands use polygamy as an excuse to pressure their wives into having more children.

*“I know it is important to discuss how many children you want. But it won’t be good for me. I would have liked to have only four children ... but then my co-wife already had four children and our husband who often says he likes children could also use it as an excuse to marry a third wife. So, I figured that telling him about my intention to stop at four children will only hurt me. I have seen it happen to other women...”* **Married woman – Nigeria** [85].

Family members’ interference was also cited by women as the reason for contraception non-use [20, 31, 44, 68, 76, 81, 82, 88–91]. In addition to the husband, the mother-in-law have an influence on women’s fertility choices as well as parents, sisters, and sisters-in-law.

*“That was our first argument with my husband about children. He was very angry when I told him about the lawlab [IUD]. [. …*]. *I already had three children, we were all living under the same roof, it was very cramped, and we had no money. But in fact, his whole family took sides against me, and all of them said we had to have more children [...]. They made it clear that if I used an IUD, I would regret it, because I might even have to leave the house and above all lose my three children.”* **Married woman, age 55 – Palestine** [92].

#### Theme 7: Religious and cultural barriers to family planning

A range of religious views towards family planning were observed across and within studies. Muslims have different religious interpretations regarding family planning, which can be categorised into two different schools of thought. One group openly accept and promote the use of contraception [81, 84, 88, 91]. While the other group strongly oppose the use of family planning, except when used for medical reasons [76, 82, 86, 88, 89, 91]. For many women, the use of family planning is believed to be permissible in Islam as long as it is used for child spacing and not limiting the number of children [46, 86, 88, 89, 91].

*“We can’t use family planning methods as we have no any right to prevent a new life from coming to the world.”* **Religious leader – Nepal** [91].

Due to the general belief held by Muslims that Islam values high fertility, many women cited wanting more children as the reason for not using any form of contraception [19–21, 24, 26, 31, 33, 39, 41, 44, 80, 81, 84, 86, 88, 91, 92].

*“Islam encourages the use of family planning without determining number of children. Our prophet Mohammed said, “Reproduce and have children as I am in the life after. I will be proud of you in front of the nations.”* **Married woman, age 39 – Jordan** [86].

The majority of Muslims share a preference for sons over daughters. Many women keep having children until they reach the desired number of sons. It is sometimes the women’s preference, which is affected by the cultural desirability for male offspring, but in some cases, it is the husband or other people in their social circle that put pressure on women to have more sons [81, 86, 88, 89, 92].

*“As an Arab woman, I have the idea that I need a son to feel “complete”. […*.] *That’s important in our society”* **Married woman, age 39 – Palestine** [92].

#### Theme 8: Marital status

Evidence suggests that unmarried women face greater difficulties accessing or obtaining contraception. These difficulties were affected by culture, family and characteristics of healthcare services provided.

Since unmarried women are believed to be sexually inactive, it is sometimes improper for them to even discuss contraception [69, 74, 76–78]. Young women expressed that they faced negative reactions when they enquired about contraception.

*“The ad was very unclear. It said to take the pill if you want a break between children. I was curious about the ad and so I asked my aunt. She scolded me and told me it was not something I should talk about.”* **Unmarried woman, age 22 – Australia** [76].

Unmarried women expressed that they lacked information and felt there was a need for formal SRH education. However, they often experienced opposition from family members when attempting to seek answers on certain sexual and reproductive matters [69, 70, 74, 76–79].

*“I don’t know anything about menstruation, how pregnancy occurs and other related issues, but I have always wanted to know more about these topics. But my family believes I don’t need to know about these subjects, as I am unmarried.”* **Unmarried woman, age 38 – Iran** [77].

In some cases, unmarried women experienced SRH related issues and were in need of medical attention. But their families seemed to dismiss their concerns or in some cases banned them from seeking healthcare services. This was mainly because they were unmarried and thus believed to not require SRH services [74, 77].

***A 38-year-old unmarried woman stated****, “I get annoyed by my delayed periods. My family tells me that, as I am not yet married, I don’t need to worry or take any measures.”* **– Iran** [77].

For other women, their mothers feared that they would lose their virginity during examinations, and would escort their daughters whenever they needed SRH related services to ensure they would not be examined [77].

Unmarried women feared stigmatization or being labelled as having premarital sexual relation if seen accessing SRH services. This fear is rooted in the cultural belief that unmarried women should not be sexually active, and many had the misconception that those services are associated with sexual activity [69, 73, 74, 77, 78].

*“If one is unmarried and has a gynaecological problem, others will think that this individual has certainly had immoral sexual relations and is probably suffering from a serious disease.”* **Unmarried woman, age 29 – Iran** [77].

Unmarried women also expressed that having services labelled as ‘sexual’ or ‘reproductive’ made it difficult and uncomfortable for them to access those services. Talking about SRH issues was often accompanied by feelings of great shame among single women [74, 77].

*“I once accompanied one of my friends to a gynaecologist’s office. I saw that all the other patients were married. Despite the fact that I needed to talk with the gynaecologist, it was difficult for me and I could not accept it. During the time I was there, I hoped that all the other patients knew I was simply accompanying my friend.”* **Unmarried woman, age 29 – Iran** [77].

Unmarried women felt more comfortable using primary care centres to receive SRH services without the fear of being exposed, as those centres offer a wide range of services not limited to SRH [73, 74, 77, 78].

*“Using public services is very good for singles. Singles are more comfortable in this way, I suppose, because they come to a centre where everyone goes; their frequent referral would not be noticed, and they would not be separated from the rest by using special labels.”* **Unmarried woman, age 29 – Iran** [74].

#### Theme 9: Access to reproductive health services

Personal, cultural and health policy factors mediated women access to reproductive health services. The economic dependency of some women on family members, either parents for unmarried women or husbands for married women, made it difficult for some to access reproductive health services when they lacked the finances to pay for the service [73, 74]. Physical and financial accessibility proved to be a facilitator to accessing reproductive health services among women. Being able to reach clinics by foot or having travel times less than 20 min, out of hours clinics or flexible hours, and low costs had positive effects on women’s health-seeking behaviours [68, 74].

*“She knows contraception. She doesn’t know how to go anyplace. No one has to go [with her]. If she gets the pill, doesn’t it finish in a month? Who buys for her? She knows [contraception]! But she can’t go. Who takes her to? She can’t go even to the maternity hospital.”* **Married woman, age 32 – Turkey** [83].

Cultural acceptability has also influenced access to reproductive health services. Married women faced difficulties accessing family planning services because they feared judgement due to the cultural unacceptability of family planning [73, 91]. Although some women believe that Islam does not forbid the use of contraception, they expressed that they cannot openly access or admit their use because it would be unacceptable.

*“Allah (God) knows everything; nothing is hidden from him. But we cannot openly share the use of FP services as others will see it negatively”* **Married woman** ***–*** **Nepal** [91].

#### Theme 10: The role of healthcare providers

Healthcare provider related factors appeared to have significantly affected women’s access to family planning services [33, 76, 80, 81, 86, 87, 89, 90]. Four issues particularly influence women’s access and use of reproductive health services: gender of the healthcare provider, paternalism, language and communication issues, and quality of the services provided.

Many women emphasized that the gender of the practitioner played a major role in whether they would accept or access the service [72, 74, 76, 87, 90]. This issue is highlighted by Islamic religious beliefs concerning modesty of women’s dress and interactions between men and women.

*“I had an appointment, the nurse got the coil ready and everything ... I went in, and she said I am going to call the [male] doctor to put the coil in for you, and I said to them, no way am I going to have a [male] doctor ... I’ll have 10 kids, but I will never have that from the doctor.”* **Married woman, age 32 – UK** [87].

Language barriers were cited in one study conducted in the UK. This made it difficult for some women to communicate with health providers and discuss their needs. Women sometimes relied on family members to interpret in health consultations, which breaches patient confidentiality, and often prevented women from discussing private issues [87].

Communication issues were also reported as barriers in family planning consultations. For example, when providers use complex language and medical terminologies, women rarely remember information given [80].

*“They told me [about the modes of action], they showed pictures. There was a book in that clinic and they showed pictures from that book. But now I do not remember, because it was very complicated”* **Married woman – Turkey** [80].

The quality of family planning services was a major barrier for some women. Healthcare providers’ misinformation, lack of skills, indifferent attitude and disrespectful treatment can lead to ineffective family planning counselling and a lack of trust [80, 81, 86, 87, 89, 90]. Some women felt judged by their doctors for their lack of control over their own fertility [86, 87, 89]. However, despite their unpleasant experiences with healthcare providers, women were not necessarily critical of providers’ behaviour [87].

*“Zaida: ‘He first shouted at me ... you have no coil, no pills you no stop children ... they [her children] making a lot of noise, they are jumping here, there ... the doctor said ... you can’t look after them two ... and how are you going to have another one now ...?’*

*Interviewer: ‘Did he upset you, the doctor?’*

*Zaida: ‘... no, no, not upset ... he is very nice.’ (Zaida, aged 34 years).”* **Married woman, age 34 – UK** [87].

The discussion of contraception may not be initiated by a healthcare provider and would not necessarily be addressed even if women ask [89, 90].

*“if they [doctors] talk more [family planning] I would benefit from them but they don’t talk, so I don’t ask.”* **Married woman – Jordan** [90].

For many women living in non-Muslim countries, having healthcare providers from similar cultural backgrounds was preferable, as they would be more understanding of their needs [72, 75]. Women felt that it facilitated conversations with healthcare providers if they share the same language, and already understand their cultural and religious beliefs and how it has an impact on their healthcare decision-making. Additionally, women felt that providers from the same culture seemed to be more caring and were keen to devote more time to their patients.

*“I heard about this doctor from Iraq and I heard that he is looking for patients. I run to him. Although I know the English language, I feel very comfortable. They understand you. If you don’t understand the English word, they can explain in Arabic. He is a man and for annual check-up, for female check-up, he himself told me that there is a doctor woman coming to our clinic on Saturdays, so he will make an appointment for me. If I go to another doctor, who doesn’t understand my religion, he will feel insulted when I tell him, sorry you cannot do this for me. Because he doesn’t understand your culture”* **Woman, marital status unknown – Canada** [72].

Studies conducted in Muslim countries showed opposing views, especially for single women. Some healthcare providers dealt with women’s concerns in an unfriendly and judgmental manner, and some women faced ridicule and negative attitudes from clinic staff and healthcare providers [72, 74, 78]. This issue was also observed in studies of healthcare professional’s views. A midwife working in a healthcare centre stated:

*“It has been established in our country that infections and gynaecological problems occur after marriage. That is, an unmarried woman cannot have such issues.”* **Midwife – Iran** [77].

Policies in some countries are preventing Muslim women from obtaining contraception [92]. For example, some healthcare providers required evidence of husbands’ consent in order prescribe contraception. Some healthcare providers explained that this was in the women’s best interest, as this would protect them from divorce [68].

*“To have the operation, I needed my husband’s signed consent. But he [the husband] refused point blank.”* **Married woman, age 47 – Palestine** [92].

#### Theme 11: Privacy and confidentiality in health services

Concern over lack of patient confidentiality was mentioned as a barrier to accessing SRH services [68, 69, 74, 76, 78]. Although women did not always seem to recognise breaches of confidentiality, the presence of a family member during consultations made it difficult to discuss the reason behind their visit [77, 88].

*“For example, one of the participant GPs who spoke Urdu had encountered a mother-in-law describing her recently married daughter-in-law as having problems becoming pregnant. When the GP discussed the issue in private, the younger woman did not want to become pregnant yet, and asked for contraceptive advice.”* **Married woman – UK** [87].

Unmarried women seemed to be concerned with healthcare providers informing family members about their visits and expressed worries regarding providers sharing information discussed during consultations. For some single women, family members often accompanied them during health visits, making it impossible for them to discuss any issues privately [74, 76–78]. Lack of privacy during consultations in some cases was the results of primary care centres failing to provide private rooms for patients [72].

A study in Egypt revealed that although patient records were stored securely in filing cabinets in the family planning clinics, a number of healthcare providers stated that they allowed family members to view women’s health records. In addition to providers’ lack of respect for confidentiality, another contributing factor is that in the Egyptian family health model, women’s medical records are a part of the combined family health records and could be easily accessed by any family member [68].

## Discussion

This is the first systematic review, to our knowledge, to synthesise the findings of quantitative and quantitative evidence on Muslim women’s SRH in a rigorous, systematic manner. There are multiple levels of factors that influence Muslim women’s SRH. Poor SRH knowledge and practices among Muslim women is a complex matter that is affected by personal, community, cultural, religious factors and existing policies and regulations. All these factors overlap and are affected by each other.

This review revealed that Muslim women studied often had little or no education and lacked awareness about family planning in general. It was also found that a significant proportion had alarming misconceptions. Lack of knowledge about contraception has been established as a significant barrier to fertility regulation. Bongaarts & Bruce found that 25% of women in 13 countries reported lack of knowledge as the primary reason for not practicing family planning [94]. In addition to poor knowledge, fear of side effects and misconceptions, particularly fear of infertility, limit women’s use of modern contraception. Some women associate condom use with disease and promiscuity, and this is true of women from different religious and cultural backgrounds [95–97].

Women’s education as well as husbands’ education positively correlates with contraceptive use [98, 99]. A systematic review of 26 demographic health surveys recognises the link between education and fertility rates [100]. Education is assumed to increase autonomy and empower women to take charge of their own fertility [100], and provide women with the knowledge to make informed decisions and use services effectively. This highlights the need to educate all women about available contraceptive methods, and how to use them.

Our review revealed that negative attitudes towards contraception influenced women’s uptake and use; these attitudes are affected by wider socio-cultural and religious factors. For example, some women believe it is against their religion to decide on how many children to have. Muslims depend on what is written in Quran and Sunnah for guidance on their day to day life. However, when it comes to family planning, there are contradictory views and interpretations of what is written. Our review also revealed that some Muslim women accept the use of contraception. According to Stephenson & Hennink (2004), psychosocial barriers were found to be the most important self-reported barrier to family planning. Psychosocial barriers were defined as religious interpretations and belief systems that limit women’s power to make decisions regarding their own fertility [101].

Shyness and modesty are major barriers that affected Muslim women’s knowledge and access to family planning and SRH services. Women expressed that they prefer discussing reproductive health matters with friends and family rather than healthcare providers, and that friends, family and the media are sources of most of their contraceptive knowledge. Women were also uncomfortable with physical examinations by physicians from the opposite gender. A preference for a physician of the same gender, particularly for gynaecological-related consultations, can be true of women from different religions [102].

The contribution of Muslim women on their own fertility choices was less significant in relation to other family member. Many married women are under a lot of pressure to stay fertile and bear children [103, 104]. A number of studies have shown that bearing children is considered essential to gain the approval of the husband’s family, protect them from divorce, and sustain economic support [95, 103–105]. Fertility decisions are mainly dominated by men and older women in the family, particularly the mother-in-law [101].

Most Muslims are known to share strong family values and patriarchal culture, which in certain situations could benefit young individuals and protect their well-being [106]. However, this can also be a barrier to women’s access to SRH information and services. Our review also revealed that unmarried Muslim women faced greater difficulties accessing SRH services compared to married women. Although both groups faced barriers accessing information and services, being unmarried by itself was a significant barrier. The social unacceptability of pre-marital sex limits young women’s SRH knowledge and access to services [95].

### Policy and research implications

The review revealed several factors that could facilitate women’s access to SRH education and services. These factors included having services labelled as general services instead of ‘reproductive’ or ‘sexual’. Women may prefer not having their identities disclosed, for example using codes instead of their names in clinics. The gender of the healthcare provider seemed to highly influence their access to services for some women. Having female doctors available or giving women the option of choosing the gender of practitioners could enhance their health seeking behaviours.

It is apparent from the results that most of provider related barriers are due to issues with the lack of policies protecting patients’ privacy and autonomy. Women highlighted the need for having their privacy respected by providers by not sharing any of their confidential information with family members and restricting family members or other people from being in the room during consultations. It is good practice for healthcare providers to announce their arrival before entering examination rooms. Enforcing policies on privacy and autonomy is likely to improve women’s access to services.

The majority of contraception studies focused exclusively on married women. Studies on unmarried Muslim women’s contraceptive knowledge, experiences, and attitudes are limited. More research is needed to explore unmarried women’s knowledge and experiences. Similarly, while this review focused on women’s perspectives, it is clear that men’s knowledge and behaviour significantly impact on women’s SRH, particularly regarding decision making, sexual relations and reproduction. As such, future research should aim to include the perspectives of men.

The findings from our review could be used to inform the development of a tailored SRH intervention that responds to Muslim women needs. Male partners can have a significant impact on women’s access to SRH education and services. Yet existing interventions mainly focus on women [107–109]. We recommend future interventions make efforts to involve male partners, with emphasis on empowering women to take charge of their own reproductive health. Many Muslims depend on guidance from religious leaders on many aspects of their lives. Having religious leaders on-board while promoting SRH education, as well as wider community involvement, is essential to ensure that SRH interventions are accepted and adopted in conservative Muslim societies.

### Limitations

Several measures have been taken to identify all relevant literature like including non-English papers, searching a broad selection of electronic databases, and screening reference lists of all included papers for additional studies. However, since we only included English and Arabic language papers, a significant proportion of the Muslim population might have been underrepresented. While it is best practice for data extraction and quality appraisal to be done independently by two reviewers, only 50% of included papers were independently appraised and extracted. However, there was an overall high level of agreement between reviewers.

There were limitations to the primary studies. A number of studies in this review lacked rigour, which could be improved by careful consideration of study design, selection of participants, and reporting of findings. We have synthesised findings from a large number of studies from different countries and settings, and whilst the specific detail differs in different settings, we have synthesised the findings within a conceptual framework to understand the factors influencing Muslim women’s access to contraception and fertility control.

Focusing on the views of people from a specific cultural or religious backgrounds can falsely create the presence of ‘issues’ for this particular group when often there are similar issues for people from different cultures and religions [110]. A review of 268 qualitative studies of factors influencing people’s SRH behaviour suggested that social forces are similar in different settings, with differences only due to the extent of the effect of these forces rather than their existence [97]. Moreover, by reporting key findings, it is easy to give the impression that a particular experience is common to an entire group, when in fact there are considerable variations both within and between every culture and religion. Arousell & Carlbom’s suggest that future research needs to move beyond simplified and generalised idea of Muslims as “one group”, to recognise religious heterogeneity and acknowledge individuals’ ability to negotiate Islamic mandates [111].

## Conclusion

There are multiple levels of factors that influence Muslim women’s reproductive health. Many women have poor reproductive health knowledge, which is a barrier to accessing contraception and other reproductive health services. Negative attitudes towards contraception and fertility control influence women’s uptake and use; these attitudes are affected by wider socio-cultural and religious factors. Sexual and reproductive health services were perceived to be for sexually active married women, and unmarried women face greater difficulties accessing reproductive health services. There is an urgent need for interventions addressing modifiable barriers to reproductive health education and services to improve knowledge, informed choice and access to services to facilitate better sexual and reproductive wellbeing for Muslim women.

## Supplementary information

**Additional file 1.** MEDLINE search strategy. This file presents a detailed search strategy for one of the databases used in this review.

**Additional file 2.** PRISMA-DTA Checklist. This file presents each PRISMA-DTA checklist item and where it is reported in the manuscript.

## Data Availability

Data sharing is not applicable to this article as no datasets were generated or analysed during the current study.

## Abbreviations

PRISMA: Preferred Reporting Items for Systematic Reviews and Meta-Analysis
SRH: Sexual reproductive health
STI: Sexually transmitted infections
UK: United Kingdom
