# Supplementary material for: Factors influencing sexual and reproductive health of Muslim women: a systematic review
Source: Reprod Health. 2020 Mar 5;17:33. doi: 10.1186/s12978-020-0888-1 (PMC7059374; doi:10.1186/s12978-020-0888-1)
Supplement: Supplementary file 1 — Additional file 1. MEDLINE search strategy. This file presents a detailed search strategy for one of the databases used in this review. [file 12978_2020_888_MOESM1_ESM.docx]

**Supplementary material 1**

# MEDLINE search strategy

Database: Epub Ahead of Print, In-Process & Other Non-Indexed Citations, Ovid MEDLINE(R) Daily and Ovid MEDLINE(R) <1946 to Present>

Search Strategy:

--------------------------------------------------------------------------------

1 Islam/ (4993)

2 islam*.tw. (4289)

3 Muslim/ (4993)

4 Muslim*.tw. (4222)

5 (arab or arabs or arabic or arabia or arabian).tw. (27492)

6 middle east/ or afghanistan/ or bahrain/ or iran/ or iraq/ or jordan/ or kuwait/ or lebanon/ or oman/ or qatar/ or saudi arabia/ or syria/ or turkey/ or united arab emirates/ or yemen/ (88185)

7 (Middle East* or Afghan* or Bahrain* or Iran* or Iraq* or Jordan* or Kuwait* or Leban* or Oman* or Qatar* or Saudi or Syria* or Turkey or Turkish or UAE or United arab emirates or Palestin* or Yemen*).tw. (140558)

8 MENA.tw. (575)

9 (middle east and north africa*).tw. (860)

10 africa, northern/ or algeria/ or egypt/ or libya/ or morocco/ or tunisia/ (33194)

11 (Algeria* or Egypt* or Libya* or Morocc* or Tunisia*).tw. (36483)

12 1 or 2 or 3 or 4 or 5 or 6 or 7 or 8 or 9 or 10 or 11 (222681)

13 exp Sexually Transmitted Diseases/ or Reproductive Health/ or Sex Education/ or Women's Health/ or Women's Health Services/ or Family Planning Services/ (412735)

14 (women* adj2 (health or healthcare)).tw. (22251)

15 ((reproductive or reproduction or sexual) adj4 (health or healthcare)).tw. (23956)

16 ((sexual or reproductive) adj2 behavio?r).tw. (24400)

17 (sex* adj2 counsel*).tw. (910)

18 ((sex* or reproduct*) adj4 educati*).tw. (17771)

19 family planning.tw. (20962)

20 exp Contraception/ (26892)

21 (Contraception or contraceptive* or birth control).tw. (69649)

22 (Sexually Transmitted Disease* or sexually transmitted infection* or sti? or std?).tw. (44799)

23 or/13-22 (543782)

24 12 and 23 (7709)

25 (wom?n* or female* or girl* or lady or ladies or wife* or wives).tw. (1939517)

26 24 and 25 (4698)

27 limit 26 to yr="2000 -Current" (2998)
